# Efficacy of incus reposition ossiculoplasty in middle ear cholesteatoma surgery

**DOI:** 10.1007/s00405-025-09755-5

**Published:** 2025-10-18

**Authors:** Shunsuke Takai, Hidetoshi Oshima, Yuri Nomura, Ryoukichi Ikeda, Yukio Katori, Toshimitsu Kobayashi

**Affiliations:** 1 Sen-En Rifu Otological Surgery Center, 2-2-108, Aobadai, Rifu-cho Miyagi-gun, Miyagi 981-0133 Japan; 2 Department of Otolaryngology-Head and Neck Surgery, Tohoku University Graduate School of Medicine, 1-1 Seiryo-machi, Aoba-ku, Sendai, Miyagi 980-8574 Japan; 3 Department of Otolaryngology-Head and Neck Surgery, Iwate Medical University, Yahaba, Morioka, Iwate, 028-3695 Japan

**Keywords:** Middle ear cholesteatoma, Tympanoplasty, Ossiculoplasty, Incus reposition technique, Hearing outcome

## Abstract

**Objective:**

Type III-r ossiculoplasty (or incus reposition) is commonly used for transmastoid decompression of the facial nerve. Although this technique should also be applicable to cholesteatoma surgery with an intact ossicular chain, it has rarely been reported. The purpose of this study was to compare the postoperative outcomes of type III-r with other ossiculoplasty techniques in middle ear cholesteatoma surgery and to evaluate the efficacy of type III-r.

**Methods:**

A retrospective chart review was performed on 227 ears (227 patients) with middle ear cholesteatoma that underwent initial surgery during the period between April 2013 and August 2023, with special reference to the 65 ears (65 patients: 41 males, 24 females, mean age 42.2 years) in which the ossicular chain was found to be continuous during surgery. Preoperative and postoperative air conduction threshold (AC) and air-bone gap (ABG) were compared between the ossiculoplasty types. Postopereative recurrence rate of cholesteatoma were compared between the ossiculoplasty types, as well.

**Results:**

The types of ossiculoplasty performed included type III-r (8 ears), type I (36 ears), type III-i (6 ears), and type III-c (15 ears). The percentage of patients with successful hearing results (ABG < 20 dB) was 100% for type III-r and type I, 100% for type III-i, and 85% for type III-c. Type III-r showed significant improvement in AC and ABG. During the follow-up period, there were no cholesteatoma recurrences in the type III-r, but 2 case recurrences (one residual and one reformation) after type I.

**Conclusion:**

In surgery for middle ear cholesteatoma with intact ossicular chain, the type III-r ossiculoplasty showed comparable hearing result with the type I. Therefore, it may be legitimate not to exclude the possibility of adopting type III-r in surgery for cholesteatoma. However, this study is preliminary and further study is necessary to reassure the lower residual recurrence of cholesteatoma after type III-r than type I.

## Introduction

Surgery is the most accepted treatment for middle ear cholesteatoma [1], and there are a considerable number of cases in which disarticulation of the intact ossicular chain is necessary depending on the location of the lesion [2]. Reconstructive ossiculoplasty is required after removal of cholesteatoma, and if an intact and mobile stapes superstructure is identified, a type III ossiculoplasty is usually performed [3].

Type III-r ossiculoplasty (or incus reposition) is an ossiculoplasty technique in which the incus is removed temporally and is repositioned at a later stage of the surgery. This technique is commonly used during transmastoid facial nerve decompression for severe facial palsy [4–13], and hearing loss is known to be a common complication [14].

There are few reports of type III-r used for middle ear cholesteatoma surgery except that of Nagashima et al. on 6 cases of cholesteatoma [15]. The purpose of this study is to compare type III-r with other ossiculoplasty techniques and to evaluate its efficacy for middle ear cholesteatoma.

## Materials and methods

### Study design/patient selection criteria

The present study protocol was approved by the Ethics Committee of Sen-En Rifu Hospital (approval number 20231208). A retrospective chart review was performed on the patients diagnosed with middle ear cholesteatoma who underwent primary surgery between April 2013 and August 2023. Twenty-two ears were congenital cholesteatoma, while the remaining 205 ears were acquired cholesteatoma (pars flaccida type 171 ears, the pars tensa type 34 ears). The following data were extracted: sex, age at surgery, surgical methods, findings of ossicular chain at surgery and a type of its reconstruction, with or without ventilation tube (VT) insertion, preoperative pure tone audiometry data closest to the operation date and those of the latest postoperative follow-up.

**Fig. 1:**
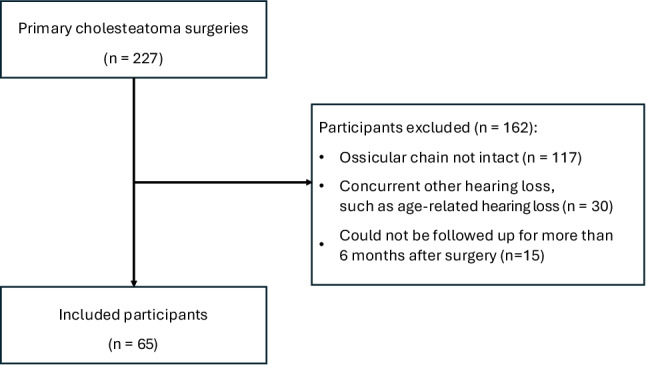
Flow chart for inclusion and exclusion of study participants

Inclusion criteria for the study were cases in which the ossicular chain was found to be continuous at the time of surgery, Cases with no other concurrent hearing loss, such as age-related hearing loss, in addition to middle ear cholesteatoma., and cases in which postoperative hearing test was performed at our hospital at least 6 months after surgery.. Inclusion and exclusion criteria are summarized (Fig. 1).

### Audiological evaluation

Audiometry was performed with a pure-tone audiometer (SA-51A; Morita Co. Ltd., Kyoto, Japan). AC and air-bone gap (ABG) thresholds were calculated as the mean of 500, 1000, 2000, and 4000 Hz, respectively. A postoperative ABG within 20 dB was considered a “successful hearing result” [16, 17]. Preoperative and postoperative AC and ABG changes were also compared.

### Surgical technique

All surgeries were primary surgeries for middle ear cholesteatoma, performed by one of the three authors (H.O., R.I., and T.K.) using the same surgical techniques at Sen-En Rifu Hospital. Most surgeries were performed using canal wall down (CWD) with soft-wall reconstruction, and the mastoid cavity was partially obliterated with bone pate. Cases with limited localized cholesteatoma were operated by exclusive transcanal technique (ETC). If preoperative evaluation revealed the presence of a sniffing habit to alleviate uncomfortable aural symptoms, ascribable to insufficiently closed Eustachian tube, and the patient was unable to stop sniffing until the day of surgery, a ventilation tube (VT) was inserted.

The ossiculoplasty techniques used in this series were classified according to the Japan Otological Society as follows [18]: type I (the original chain was preserved), type III-r (repositioning of the incus removed at an earlier stage of surgery), type III-i (interposition between the handle of the malleus and the stapes head using material such as a remodeled ossicle, a piece of cartilage), type III-c (columella made of either ossicle or cartilage, placed between the tympanic membrane and the stapes head), type IV (columella made of either ossicles or cartilage, placed between the tympanic membrane and the stapes footplate), and WO (without reconstruction of the ossicular chain).

**Fig. 2:**
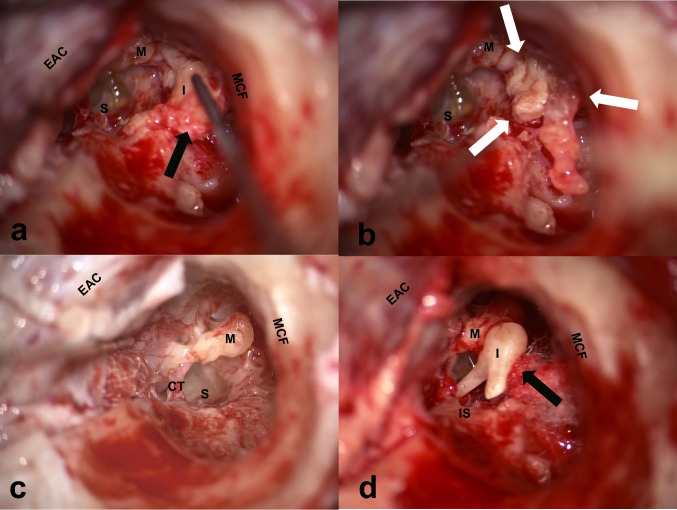
Operative findings in the incus repositioning technique (type III-r). **A** The incus surrounded by cholesteatoma matrix (black arrow) was removed temporally. **B** After removal of the incus, the cholesteatoma in the attic (white arrow) is clearly visible. **C** After removal of the cholesteatoma. **D** The cleaned incus was repositioned while supported by a piece of cotton ball (black arrow). Fibrin glue was used to stabilize the reconstructed ossicular chain. M: malleus, I: incus, S: stapes, IS: incudostapedial joint, CT: chorda tympani nerve, MCF: middle cranial fossa, EAC: external auditory canal

The type of ossiculoplasty selected depended on the surgeon’s judgment at the time of surgery. In general, type III-r ossiculoplasty was chosen over type I when the cholesteatoma lesion extended to an area that was difficult to eradicate while maintaining an intact ossicular chain. This included the medial side of the incus body and the area anterior or medial to the head of the malleus. Type III-r was also chosen over Type I when the cholesteatoma matrix was firmly attached to the lateral surface of the head of the malleus or the body of the incus. To reduce the risk of irreversible damage to the inner ear during lesion cleaning, the incus was temporarily removed. The removed incus was examined and cleaned under a microscope to determine its suitability for reuse. If the incus body was significantly eroded or if matrix remnants were present, a type III-i or III-c procedure was performed using a remodeled incus or a piece of cartilage. If the malleus head was substantially eroded, it was removed and a type III-i or III-c procedure was performed using a remodeled ossicle or a piece of cartilage. A minimal amount of fibrin glue was used to stabilize the reconstructed ossicular chain. A representative operative findings of a typical type III-r ossiculoplasty case are shown (Fig. 2).

### Statistical analysis

Statistical analysis was performed using GraphPad Prism 7.02 (GraphPad Prism Software Inc., San Diego, California, USA). The Shapiro-Wilk normality test was used to determine whether continuous variables had a parametric distribution. The Wilcoxon signed-rank sum test, Wilcoxon rank sum test, and Kruskal-Wallis test were then used to compare continuous variables with nonparametric distributions. *P* value <0.05 was considered statistically significant.

## Results

### Main case characteristics

A total of 162 ears were excluded from the study because they did not meet the inclusion criteria (Fig. 1). In the analysis described below, the participants included 65 patients (41 males and 24 females, mean age 42.2 ± 15.9 years, range 6–76 years) with 4 patients (6.2%) younger than 18 years (Table 1). The ossiculoplasty technique used in the 65 ears were type III-r in 8 ears, type I in 36 ears, type III-i in 5 ears, and type III-c in 15 ears. Six type I ears underwent ETC, and the remaining 59 ears underwent CWD with soft-wall reconstruction and partial obliteration of the mastoid cavity.

**Table 1:** Characteristics of the patients meeting the inclusion criteria

All cases (*n* = 65)	All cases (*n* = 65)	Type III-r (*n* = 8)	Type I (*n* = 36)	Type III-i (*n* = 6)	Type III-c (*n* = 15)
Gender; male/female	41/24	5/3	20/16	5/1	11/4
Age (year)	42.2 ± 15.9	37.6 ± 10.1	41.6 ± 16.4	42.3 ± 8.6	44.2 ± 17.5
Pathological classification					
Pars flaccida	61	8	36	5	12
Pars tensa	2	0	0	1	1
Congenital	2	0	0	0	2
Percentage of ABG assessed.	54 (83%)	7 (88%)	29 (81%)	5 (83%)	13 (87%)
VT placement Rate	22 (34%)	3 (38%)	14 (39%)	2 (33%)	3 (20%)
Follow-up period (year)	1.8 ± 0.9	1.5 ± 0.9	1.7 ± 0.7	2.2 ± 1.7	1.9 ± 0.9
Recurrence Rate	5 (7.7%)	0 (0%)	2 (5.6%)	1 (8.3%)	2 (13%)
Due to residual			1	1	1
Due to retraction			1		1

*ABG* Air Bone Gap, *VT* Ventilation tube

Data are presented as mean ± standard deviation (SD) or percentage

Patients who underwent VT because of failure to stop habitual sniffing were type III-r in 3 ears (38%), type I in 14 ears (39%), type III-i in 2 ears (33%), and type III-c in 3 ears (20%) (Table 1). There were no cases of VT inserted after surgery. At present, postoperative cholesteatoma recurrence rate were type III-r in 0 ears, type I in 2 ears (one each due to residual and retraction), type III-i in 1 ear (due to residual), and type III-c in 2 ears (one each due to residual and retraction) (Table 1).

### Hearing outcomes after ossiculoplasty

**Fig. 3:**
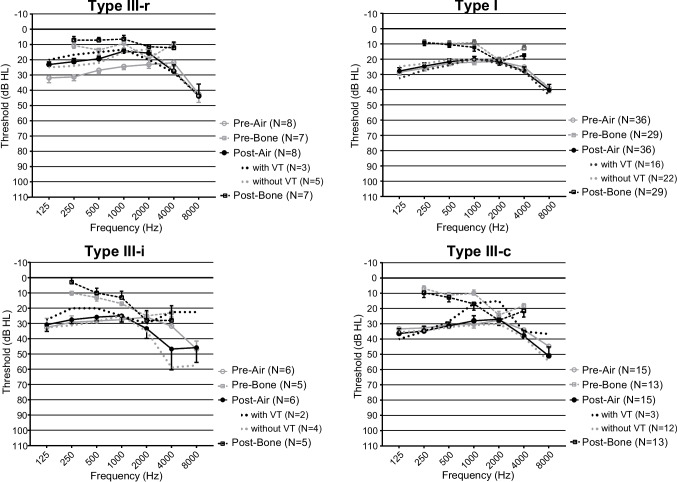
Mean frequency-specific hearing levels pre- and postoperatively for air and bone conduction for each ossiculoplasty. Each audiogram shows the frequency-specific preoperative and postoperative audiometric data with ossiculoplasty type III-r, type I, type III-i, and type III-c. Circles (solid line) and squares (dashed line) represent air and bone conduction threshold, and the gray and black lines represent pre- and postoperative hearing levels, respectively. Mean frequency-specific hearing levels for postoperative air conduction with/without ventilation tube (VT) placement are represented by dotted black and dotted gray lines, respectively

In the 65 enrolled ears, the pre- and postoperative audiograms for each ossiculoplasty technique are shown (Fig. 3). In 54 of 65 ears (83%), the ABG could be obtained as a result of pre- and postoperative BC hearing measurements (Table 1). For pre- and post-operative AC and ABG, a Dunn post-hoc test for Kruskal-Wallis test showed a statistically significant difference only between type I and type III-c for pre-operative AC.

Postoperative hearing outcome assessment was performed at the following time intervals after surgery: type III-r 1.8 years postoperative (SD 0.9 years, min 7 months, max 4 years), type I was 1.5 years postoperative (SD 0.9 years, min 7 months, max 3 years), type III-i 2.2 years (SD 1.7 years, min 9 months, max 6 years), and type III-c was 1.9 years postoperative (SD 0.9 years, min 9 months, max 4 years) (Table 1).

**Fig. 4:**
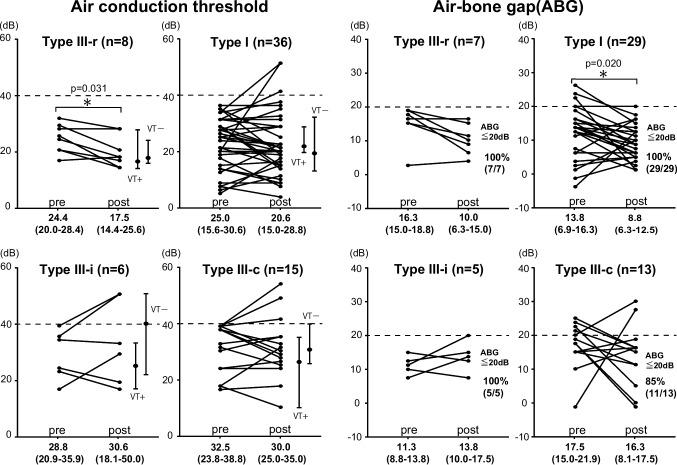
Air conduction threshold (AC) and air-bone gap (ABG) at pre-surgery and post-surgery (0.5, 1, 2,4 kHz) for each ossiculoplasty. Wilcoxon signed-rank sum test was performed to evaluate significant differences between preoperative and postoperative hearing outcomes and the use of VT placement. Hearing outcomes are presented as median (interquartile range)

Postoperative AC and postoperative ABG are shown (Fig. 4). The percentage of patients with postoperative ABG less than 20 dB and “successful hearing result” [19, 20] was 100% (7/7 ears) for type III-r, 100% (29/29 ears) for type-I, 100% (5/5 ears) for type III-i, and 85% (11/13 ears) for type III-c (Fig. 4).

After surgery for middle ear cholesteatoma with continuous ossicular chain before surgery, type III-r had significantly improved AC (preoperative; 24.4 (20.0–28.4) dB, postoperative; 17.5 (14.4–25.6) dB, *p* = 0.031, Wilcoxon signed-rank sum test) (Fig. 4). On the other hand, using the other ossiculoplasty technique, only the ABG of type I ossiculoplasty improved significantly (preoperative; 13.8 (6.9–16.3) dB, postoperative; 8.8 (6.3–12.5) dB, *p* = 0.020, Wilcoxon signed-rank sum test), but otherwise there was no statistically significant difference in either AC or ABG before or after surgery (Fig. 4).

Postoperative AC in ears with and without VT placement is indicated by the dotted line (Fig. 3). In particular, in cases where type I was performed, cases with VT showed a tendency to increase thresholds at lower frequencies, such as 0.25 and 0.5 kHz, compared to cases without VT. However, there was no statistically significant difference in postoperative AC in any of the ears regardless of VT placement (Fig. 4).

## Discussion

### Theoretically predicted postoperative air-bone gap after the type III-r estimated from normal cadaveric human temporal bone study

Hato et al. reported that the posterior incudal ligament and buttress do not play a significant role in the acoustic function of the ossicles based on experiments on human cadaveric temporal bones using a laser Doppler vibrometer system [21]. The effect of sacrificing the posterior incudal ligament, which is unavoidable in type III-r, on hearing is negligible. They also performed another experiment on the type III-r using 11 fresh human temporal bones with the same methods [22]. After the baseline measurements, the incus was carefully removed and reattached to the normal position with cyanoacrylate glue. Footplate displacement was approximately 7 dB less than in the intact middle ear at frequencies below 1 kHz. They concluded that in middle ear surgery, the surgical exposure could be improved by resection of the incus, because the acoustic damage of the incus repositioning was less than 6 dB on average in the mid-frequency range. Alternatively, it can also be interpreted that a decrease in hearing loss of about 6 dB is inevitable even after a carefully performed type III-r.

### Type III-r during facial nerve decompression

Although there have been few reports of type III-r being used in middle ear surgery, it has been most commonly used in facial nerve decompression. May & Klein reported that 14% of 43 patients had an air-bone gap of 15-dB or more after intratemporal facial nerve surgery with temporal incus removal and repositioning (analogous to type III-r), and that the resulting hearing loss was a major complication that should be included in the preoperative informed consent, as should the risk of sensorineural hearing loss due to the noise from surgical instruments [14]. Inagaki et al. reported the hearing outcomes of 108 cases of facial nerve decompression including 14 cases in which the temporal incus removal and repositioning technique (analogous to type III-r) was required to access the horizontal portion of the facial nerve due to anatomical infeasibility such as the thick incus body [2]. An increase of 12.7 dB in the mean air conduction threshold (0.5, 1, 2, 4 kHz) was observed in this group and air-bone gap greater than 20 dB was found in one case. Inagaki et al. stated that the difficulty in achieving good ossicular coupling may underlie this hearing outcome. They concluded that the ossicular chain preservation technique has an advantage over incus removal and reconstruction techniques (analogous to type III-r) in preserving hearing [2]. Kumai et al. reported 25 cases of delayed facial nerve decompression for Ramsey-Hunt syndrome. They used the incus repositioning technique (analogous to type III-r) and found a change in baseline hearing thresholds from an average of 18.0 ± 9.6 dB preoperatively to 25.0 ± 12.1 dB postoperatively, although the difference was not statistically significant (*p* =.067) [19]. Kumai’s report is important because it showed that the increase in average hearing threshold after the type III-r in living human ears (7 dB) can be close to the theoretical predicted value (6 dB) obtained from the human temporal bone study [22].

### Type III-r in cholesteatoma surgery: Previous reports

The reports by Hato et al. and Kumai et al. encourage the use of type III-r in clinical cases [19, 21, 22]. However, its use in ears with middle ear pathology requires additional consideration of factors including the ventilation of the middle ear system and Eustachian tube dysfunction, as well as longer follow-up. Nagashima et al. reviewed the surgical outcomes of 66 patients with pars flaccida cholesteatoma who underwent ossicular chain-preserving tympanoplasty [15]. Six cases received type III-r, and postoperative ABG was within 20 dB (success) in all cases at one year after surgery, which is favorably comparable with the outcome of 9 cases using type I performed by them. However, the success rate of the type III-r decreased at 5 years after surgery. They also compared their data of type III-r with those of type III-c or type III-i in cholesteatoma surgery and argued against the use of type III-r if good middle ear ventilation is not expected postoperatively.

### Type III-r in cholesteatoma surgery: The present outcomes

In the present study, the average ABG after type III-r was 10.0 dB. This is slightly higher than the theoretical predicted value of 6 dB in Hato’s temporal bone experiment [22], but similar to the average hearing threshold shift of 7 dB in the facial nerve decompression reported by Kumai et al. [19]. The present outcomes seem satisfactory because the subjects had pathological ears with a mean preoperative ABG of 16.3 dB, presumably due to the pathology around the ossicles and/or the tympanic membrane. Hearing improved by approximately 6 dB postoperatively, probably because pathology limiting ossicular mobility, such as cholesteatoma, granulations, and calcifications, could be meticulously cleaned by adopting the type III-r.

### Advantages of type III -r over type I

Type I and type III -r inherit the same risk of cholesteatoma recurrence using the ossicles to which the cholesteatoma was attached. However, no recurrence has been found in cases after type III-r until now, whereas two recurrences (2/40 (5%)) were found after type I in our study. The two recurrences were due to residual and retraction, respectively. Certainly, recurrence due to retraction may be caused by Eustachian tube dysfunction, but Nagashima also reported that the recurrence rate after the type III-r was lower than that of type I [15]. Judging the suitability of ossicles for reuse is not difficult when performed under microscopic observation outside the operating field. If the judgment is that the type III-r is not feasible, other methods of ossiculoplasty (type III-c or type III-i) should be selected. In addition, the type III-r has an advantage over the type I in that it reduces the risk of damage to the inner ear during removal of the cholesteatoma matrix from the ossicles.

### Requirement for successful type III-r

Applicability of type III-r should be carefully determined based on the location and nature of the cholesteatoma matrix, the conditions of the ossicles, mucosa and Eustachian tube function. The mobile malleus and stapes with usable articular facets, ventilated attic supported by the normal Eustachian tube function are the prerequisites for good hearing outcomes after the type III-r. It is now well known that there are cases of habitual sniffing in cholesteatoma patients due to an insufficiently closed Eustachian tube. In such patients who were unable to stop habitual sniffing despite preoperative instructions, a VT was inserted through the tympanic membrane. This is because habitual sniffing, if continued, induces a high negative pressure in the middle ear, which carries a high risk of retraction of the reconstructed tympanic membrane and postoperative recurrence of cholesteatoma [20, 23–28]. In the present series, a VT was used in 22 ears with suspected impaired ET function. The VT placement can cause low frequency hearing loss at 0.25 kHz and 0.5 kHz [29]. In our study, in type I cases, thresholds at lower frequencies tended to be elevated in cases with VT placement compared to those without VT placement, as reported previously. However, there was no statistically significant difference in mean postoperative AC.

### Tips of the type III-r

The surgical tips of type III-r, in our opinion, is to get a good view of both joints in one operating field, and the incus is supported with a small piece of cotton ball temporarily placed under the body of the incus during manipulation, and joints are glued with a minimum amount of fibrin glue. After the glue is fixed, the movement of the whole ossicular chain is carefully examined to ensure a good coupling of the ossicles [2, 15, 21, 22]. As we used the canal wall down technique in the present subjects, it provided a good view of the incudomalleolar and incudostapedial joints in the same operative field, which was helpful for precise repositioning of the incus.

### Suggested new policy in selecting the type of ossiculoplasty for cholesteatoma

Although the total number of 8 ears (4% of the total cholesteatomas and 15% of which the ossicular chain was preserved) is small, the present results may suggest new policy in the selection of the type of ossiculoplasty. Since the postoperative hearing outcomes of the type III-r were comparable to those of type I, the type III-r could be considered for cases involving advanced extension around the ossicles. Furthermore, the previous report and the present study results suggest that type III-r may have a lower recurrence rate than type I, which may be an advantage in choosing type III-r. Additionally, type III-r should be more frequently considered, even for cases in which type III-c or type III-i has been conventionally selected, when erosion of the incus is mild.

### Limitations of the present study

One limitation in the present study is that only cases in which the ossicular chain remained intact were included, which may limit the generalizability of the findings to more complex cases requiring complete ossicular reconstruction. That is, we cannot exclude the possibility that the extension of the cholesteatoma, the preoperative air-bone gap, the destruction of the malleus, and the intraoperative judgement by the operators may have influenced the decision to perform type III-r, thus introducing a possible confounding effect. In addition, the follow-up period of at least 6 months may not be sufficient to assess long-term hearing stability. Therefore, it is important to continue follow-up. Finally, the present study had a limited number of cases, especially for type III-r (8), which may not be sufficient for clinical generalization due to the small sample size. Therefore, it is important to confirm the study’s suggestion that type III-r results in lower residual cholesteatoma recurrence than type I by increasing the number of cases.

## Conclusion

In middle ear cholesteatoma surgery with an intact ossicular chain, type III-r ossiculoplasty showed comparable hearing results to type I. Therefore, it is reasonable to consider using type III-r in cholesteatoma surgery, even though it has been reported far less frequently than in facial nerve decompression surgery. However, the present study is preliminary, and further research is necessary to confirm the results with a larger sample size.
